# Comparison of Machine Learning Models for Hazardous Gas Dispersion Prediction in Field Cases

**DOI:** 10.3390/ijerph15071450

**Published:** 2018-07-10

**Authors:** Rongxiao Wang, Bin Chen, Sihang Qiu, Zhengqiu Zhu, Yiduo Wang, Yiping Wang, Xiaogang Qiu

**Affiliations:** 1 College of System Engineering, National University of Defense Technology, 109 Deya Road, Changsha 410073, China; wangrx-nudt@foxmail.com (R.W.); s.qiu-1@tudelft.nl (S.Q.); admin@steven-zhu.me (Z.Z.); will_king163@163.com (Y.W.); michael.qiu@139.com (X.Q.); 2 Faculty of Electrical Engineering, Mathematics and Computer Science, Delft University of Technology, Building 28, Van Mourik Broekmanweg 6, 2628 XE Delft, The Netherlands; 3 The Naval 902 Factory, Shanghai 200083, China; foolwangrain@126.com

**Keywords:** hazardous gas dispersion prediction, back propagation network, support vector regression, input selection, field case

## Abstract

Dispersion prediction plays a significant role in the management and emergency response to hazardous gas emissions and accidental leaks. Compared with conventional atmospheric dispersion models, machine leaning (ML) models have both high accuracy and efficiency in terms of prediction, especially in field cases. However, selection of model type and the inputs of the ML model are still essential problems. To address this issue, two ML models (i.e., the back propagation (BP) network and support vector regression (SVR) with different input selections (i.e., original monitoring parameters and integrated Gaussian parameters) are proposed in this paper. To compare the performances of presented ML models in field cases, these models are evaluated using the Prairie Grass and Indianapolis field data sets. The influence of the training set scale on the performances of ML models is analyzed as well. Results demonstrate that the integrated Gaussian parameters indeed improve the prediction accuracy in the Prairie Grass case. However, they do not make much difference in the Indianapolis case due to their inadaptability to the complex terrain conditions. In addition, it can be summarized that the SVR shows better generalization ability with relatively small training sets, but tends to under-fit the training data. In contrast, the BP network has a stronger fitting ability, but sometimes suffers from an over-fitting problem. As a result, the model and input selection presented in this paper will be of great help to environmental and public health protection in real applications.

## 1. Introduction

Hazardous gas emissions and leaks pose important threats to air quality and public health. For instance, the methyl isocyanate leak accident in Bhopal (1984) caused thousands of deaths [1]. Meanwhile, the airborne contaminants released from industrial areas also have an adverse impact on the lives of nearby residents. Consequently, gas emissions and accidental leaks have been attracting increasing attention in recent years. Considering the aforementioned issues about hazardous gases, predicting their atmospheric dispersion is of great value. Based on the predicted concentration distribution, managers are able to not only evaluate the harm of hazardous gas to human health, but also develop evacuation plans more responsibly.

The atmospheric dispersion (ADS) model is widely applied to predict the transportation and dispersion of gas in air. There have been many effective models for predicting gas dispersion. Conventional ADS models can be roughly categorized in three main types: the Gaussian model [2,3], the Lagrangian stochastic (LS) model [4,5], and the computational fluid dynamics (CFD) model [6,7]. The Gaussian model is the most widely used model in atmospheric dispersion prediction. Requiring only a few input parameters, this model uses a simple expression with fast computing. However, built on the ideal dispersion environment, the Gaussian model takes few terrain conditions into consideration. Therefore, this model is not accurate enough in some complex environment conditions (e.g., urban areas with complex topography). The LS model uses a stochastic method, and describes the gas transport as a Markov process with a number of particles. In contrast, the CFD model is based on sophisticated fluid dynamics equations [7]. Compared with Gaussian models, these two models are usually more accurate but less efficient for atmospheric dispersion prediction. Their higher computational costs limit their applications in emergency response to gas leak accidents. Therefore, there is a need for an atmospheric dispersion model with both high accuracy and acceptable efficiency.

To address this problem, many researchers have introduced machine learning (ML) models into atmospheric dispersion prediction, such as the artificial neural network (ANN) [8,9,10,11,12] and support vector regression (SVR) [13,14,15,16]. ML models usually have an excellent capacity for predicting the complex relationship between the input and output [17,18]. Trained by some pre-determined dispersion scenarios, these models tend to obtain relatively high prediction accuracy for these scenarios. Moreover, the computation of trained ANN or SVR prediction models is relatively fast. Among various types of ANN, the back propagation (BP) network is most widely used to predict atmospheric dispersion. Compared with other ANNs like the radial basis function (RBF) network, the BP network has fewer hyperparameters to determine [19]. Therefore, the BP network is relatively easy to build and train. Boznar et al. [8] developed a neural network-based method to predict the $SO_{2}$ pollution around a thermal power plant in Slovenia, and acquired promising results. Pelliccioni [9] developed an integrated model for air pollution dispersion prediction. This model filtered the concentration produced by the Gaussian dispersion model with a neural network, and consequently improved the prediction accuracy of the virtual height dispersion model (VHDM) and the skewed puff model (SPM). As for the SVR, it is derived from the support vector machine (SVM) [13] and inherits the beneficial properties of the SVM, such as good performance for small-scale data. Yeganeh et al. [14] used the combination of SVR as a predictor and partial least squares (PLS) as a data selection tool to predict daily CO concentrations. The results demonstrated that the hybrid PLS-SVR model is quicker and more accurate than the SVR model.

However, most of ML models in the aforementioned research are directly constructed on the inputs of some original monitoring parameters. With these inputs, the ML models usually yield acceptable prediction results. However, this selection of input parameters possibly increases the difficulty in model training and consequently reduces the prediction accuracy, because the relationship between the original monitoring parameters and the output (concentration) is usually quite complex. Therefore, although these ML models were tested successfully in the research mentioned above, they can be further improved by the more effective input selection. In addition, existing research usually focuses on one particular ML model, instead of comparing different ML models in gas dispersion prediction and giving some guidance on model selection. In fact, different ML models vary greatly in terms of performance, for example in fitting and generalization ability. Moreover, trained by pre-determined scenarios and tested by particular cases, the performances of the ML model heavily depend on the training set and test set. Therefore, the influence of the sizes of training set and test set on the prediction performance should be analyzed. This analysis also helps to further reveal the difference of ML models on the fitting and generalization abilities.

In this paper, two machine learning models (i.e., the BP network and SVR model) are respectively involved and applied in the hazardous gas dispersion prediction. To improve the prediction performance of the ML models with original input parameters, the Gaussian integrated parameters are formed and used as the inputs. To comprehensively compare the performances of proposed models with different input selections, they are firstly tested and evaluated on two field data sets (Project Prairie Grass [20] and Indianapolis [21,22]) with different terrain conditions. Next, by varying the sizes of the training set and test set, these ML models are further evaluated and compared. Based on the results, the fitting and generalization abilities of these two ML models are discussed, which is followed by some guidance for model selection.

The rest of this paper is organized as follows. Section 2 describes the two field data sets and structures of the BP network and SVR model for prediction, as well as the input selection. The performances of these ML prediction models are tested on two field cases in Section 3. The discussion is given in Section 4, followed by the conclusions in Section 5.

## 2. Materials and Methods 

### 2.1. Brief Desciption of the Field Data Sets

#### 2.1.1. Project Prairie Grass Data Set

The Prairie Grass data set is a well-known field experimental data set referring to a typical hazardous gas emission case with flat terrain and low stack emission. This tracer experiment was carried out in an open country in O’Neil, NE (USA, 42.493° N and 98.572° W) from July to August, 1956. The sulfur dioxide ($SO_{2}$) tracer was released from a continuous point source at the height of 0.46 m without buoyancy. Concentration data were collected by five semi-circular arcs (50, 100, 200, 400, 800 m downwind of the release) of receptors. All receptors over the 180-degree arcs had the same height of 1.5 m. They were centered on the emission source. The receptor spacing was two degrees on the inner four arcs, and one degree on the outer arc of 800 m. As for the meteorological data, the horizontal mean wind direction and wind speed were collected at two locations (i.e., (a) 25 m west of the release source, and (b) 450 downwind of the source and 30 m west of the centerline of the receptor array) for two periods (i.e., 10 min and 20 min). Other meteorological data (e.g., air temperature) were observed by the meteorological tower and rawinsonde. There are 68 releases containing tracer data (6888 valid samples used in this paper) and meteorological data in the data set.

#### 2.1.2. Indianapolis Data Set

The Indianapolis tracer experiment was conducted in a typical urban area of Indianapolis, Indiana, USA, from 16 September to 11 October 1985. In this experiment, the source was an 83.8 m stack (with diameter 4.72 m) at the Perry K power plant in Indianapolis, which released the $SF_{6}$ tracer. The geographic coordinates of this stack were UTM-N (Universal Transverse Mercator) 4401.59 km (39.8° E latitude) and UTM-E 571.40 km (86.2° E longitude). In contrast to the Prairie Grass field experiment, the Indianapolis experiment was carried out under complex terrain conditions. There were many buildings within one or two kilometers of the source stack. These buildings can influence the tracer dispersion in air significantly, which is a challenge to the accurate concentration prediction. As for the experimental data, more than 100 h of tracer concentration data in 17 days were used here, as well as the meteorological data covering all atmospheric stability classes and most common wind direction and speed ranges. The tracer concentrations were measured by a network including about 160 ground-level sensors on several semi-circular arcs, at distances ranging from 0.25 to 12.0 km from the source stack. Therefore, the range of the monitoring distance was about 12 km. The unit of the tracer data is ppt (volume fraction: one billionth). Data were taken in subsets of 8 or 9 h each day. In total there are 17 such subsets used in our work, representing the data of different days and containing tracer data (23,900 valid samples used in our work) and meteorological data.

### 2.2. Back Propagation Network

**Figure 1:**
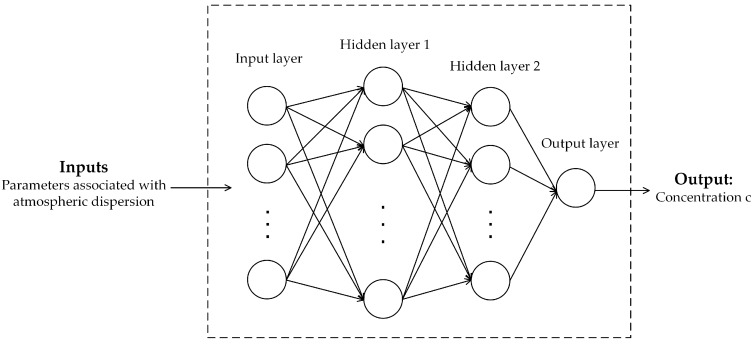
The structure of the back propagation (BP) fitting network for prediction.

The artificial neural network (ANN) is a most widely applied ML model in dispersion prediction. Because of its excellent fitting ability, the ANN is able to approximate complex nonlinear function. As for the computational efficiency, a trained ANN can compute the predictions rapidly. In this paper, the back propagation (BP) fitting network is built to predict the concentration of interest points. This type of network has been quite popular in the research of dispersion prediction [8,9]. The structure of the fitting BP network is shown in Figure 1. To achieve higher prediction accuracy, two hidden layers are applied here. The inputs of the network are usually parameters associated with atmospheric dispersion. These parameters usually include the meteorological parameters, the parameters related to the points of interest, and the source terms. The details of input selection will be discussed in Section 2.4. These inputs should be selected carefully for better performance. The selection of input will be discussed in Section 2.4. As for the activation units, the activation functions of all hidden layers are “tansig” for better convergence speed and solution accuracy [23]. The “tansig” is a kind of sigmoid function with the expression: $f(x)=\frac{2}{1+e^{-2x}}-1$. In contrast, the activation function of the output layer (only one neuron) is “purelin” to output the continuous value of concentration. Further, the neuron numbers of two hidden layers, which are critical parameters for the network, should be adjusted according to the performance of the BP network. With appropriate neuron numbers of hidden layers, the BP network can perform well on both accuracy and the convergence speed. In our work, the BP network is trained by the MATLAB neural network toolbox. The detailed process of the model training and the optimization of the neuron numbers will be introduced in the Section 3.1.

### 2.3. Support VectorRegression

Support vector regression (SVR) is an extension of the support vector machine (SVM) developed by Vapnik [13] to solve the regression problem. The idea of SVR is based on the linear regression function in a high-dimensional feature space, where the input data is mapped via a kernel function. In addition, instead of minimizing the training error, the SVR attempts to minimize the generalization error bound to achieve better generalization. Given a set of training points $\left\{(x_{1},z_{1}),\ldots ,(x_{l},z_{l})\right\}$ where $x_{i}\in R^{n}$ is an input and $z_{i}\in R^{1}$ is a target output, the standard form of SVR can be expressed as:

(1) $$\begin{array}{ll} \underset{\omega ,b,\xi ,\xi^{*}}{\min} & \frac{1}{2}\omega^{T}\omega +C\sum_{i=1}^{l}\xi_{i}+C\sum_{i=1}^{l}\xi_{{}_{i}}^{*}\\ s.t. & \omega^{T}\phi (x_{i})+b-z_{i}\leq \varepsilon +\xi_{i},\\ & z_{i}-\omega^{T}\phi (x_{i})-b\leq \varepsilon +\xi_{{}_{i}}^{*},\\ & \xi_{i},\;\xi_{{}_{i}}^{*}\geq 0,i=1,\ldots ,l, \end{array}$$

where C is the regularization parameter and ε is the error tolerance. ξ and $\xi^{*}$ are the lower and upper slack variables, respectively. The ω and b are the parameters of the linear regression model in the high dimensional feature space. The goal of SVR is to determine the optimized ω and b, and get the regression model. The approximate solution function is:

(2) $$y(x)=\sum_{i=1}^{l}(-\alpha_{i}+\alpha_{i}^{*})K(x_{i},x)+b$$

where $K(x_{i},x_{j})=\phi {\left(x_{i}\right)}^{T}\phi (x_{j})$ is the kernel function and α is the support vector. Here, the radial basis function (RBF), which is widely used in the SVR, is selected as the kernel function. The output of SVR is the concentration of the interest point, and the selection of the input will be introduced in Section 2.4. To build an optimization SVR model, the tolerance ε, regularization parameter C and the spread parameter σ in the RBF function should be carefully selected (Section 3.1). The Library for Support Vector Machines (LIBSVM) [24] is applied here to build the SVR model.

### 2.4. Selection of the Input Parameters

The monitoring data in the Prairie Grass and Indianapolis field experiments are used to build the BP network and SVR model for prediction. For example, some common original monitoring parameters of Prairie Grass data set are displayed in the Table 1. Selecting all the parameters as the inputs of ML models is impractical, because some of these parameters do not contribute greatly to the gas concentration. Using these redundant parameters barely improves the accuracy of prediction, and increases the difficulty of training. Therefore, only $D_{x},\;D_{y},\;Q,\;V,\;Dir,\;STA,\;T,\;H_{s}$ are selected here. These parameters are the main factors affecting gas dispersion and are easy to obtain from the data set. In addition, these selected parameters are the inputs of many typical atmospheric dispersion models, like the Gaussian model. The target height Z is not included because it is fixed in the data set. These parameters are also available in Indianapolis data set. Therefore, the aforementioned parameters are used as inputs in the two field cases.

Based on these original input parameters, a ML prediction model can be constructed. It should be noted that there are four different observations of wind direction and wind speed observed (i.e., by two stations for the 10-min and 20-min periods) in the Prairie Grass case, as mentioned in Section 2.1. These wind field parameters are all used to build the ML model, and to generate four values of downwind distance $D_{x}$ and crosswind distance $D_{y}$. Therefore, the input vector of the ML model has 20 elements. With regard to the Indianapolis data set, there are also four observations of wind speed and direction available. Therefore, the ML models constructed on the original monitoring parameters of the Indianapolis field data set also have 20 input parameters.

However, it may be still difficult to train the ML model with these original parameters because the features of the atmospheric dispersion should be extracted from these original parameters before the training process. Therefore, the integrated Gaussian parameters are considered. The Gaussian model is the most widely used atmospheric dispersion model and the results are trustworthy for near-field dispersion cases. The integrated Gaussian parameters, which are from the Gaussian plume model, are expressed as:

(3) $$\left\{ \begin{array}{l} G_{y}=\;exp(-\frac{D_{y}^{2}}{2\sigma_{y}^{2}})\\ G_{z}=\;exp[-\frac{{\left(z+H_{s}\right)}^{2}}{2\sigma_{z}^{2}}]+exp[-\frac{{\left(z-H_{s}\right)}^{2}}{2\sigma_{z}^{2}}] \end{array}\right.$$

where $G_{y}$ and $G_{z}$ represent the Gaussian parameters in the crosswind and vertical directions, respectively. $D_{y}$ is the crosswind distance. z and $H_{s}$ describe the heights of the interest point and emission source, respectively. $\sigma_{y}$ and $\sigma_{z}$ are the standard deviations that determine the Gaussian distributions in the crosswind and vertical directions. The two standard deviations can be calculated by:

(4) $$\left\{ \begin{array}{l} \sigma_{y}=\;a\cdot D_{x}^{b}\\ \sigma_{z}=\;c\cdot D_{x}^{d} \end{array}\right.$$

where $D_{x}$ is the downwind distance. a,b,c and d are dispersion coefficients derived from the atmospheric stability class STA [25] according to Vogt’s scheme [26]. To generate the input vector, the integrated Gaussian parameters are firstly derived from the original parameters, and then combined with Q,V,Dir,T, and STA. The wind speed V and wind direction Dir both have four observed values. Consequently, four different $G_{y}$ and $G_{z}$ are generated accordingly. Therefore, the input vector also includes 20 elements.

## 3. Application: Prairie Grass and Indianapolis Field Case Study

In this section, the proposed BP network and SVR models for prediction with different types of input selections are all tested on the Prairie Grass and Indianapolis data sets, shown as follows:

- BP network with original input parameters;
- BP network with integrated Gaussian input parameters;
- SVR with original input parameters;
- SVR with integrated Gaussian input parameters.

The characteristics of BP network and SVR as well as the influence of different input parameters can be analyzed by evaluating their prediction performances. In addition, the fitting and generalization abilities of the two ML models are further discussed by evaluating their prediction performances on the varying-sized training set and test set.

### 3.1. Configurations and Results of the Machine Learning Models

According to the structures introduced in Section 2, the ML models are constructed on the two field data sets, respectively. Firstly, the tracer data and original monitoring parameters are extracted from the data set. Then, the 68 releases in the Prairie Grass data set are randomly divided into 60 releases for training and validation (6239 samples in total) and 8 releases (649 samples) for testing. As for the Indianapolis case, the tracer and meteorological data from 20 September to 11 October (15 days) were used for training and validation (21,276 samples in total) while the data from 17 and 19 September were used for testing (2624 samples). The statistical indicators of the input parameters used in the two field cases are shown in Appendix A.

#### 3.1.1. BP Network with Original Monitoring Input Parameters

The selected original monitoring parameters $D_{x},\;D_{y},\;Q,\;V,\;Dir,\;STA,\;T,\;$ and $H_{s}$ are firstly normalized to (0, 1) and then used to form the input vector. Afterwards, the training process of the BP network is conducted by the MATLAB neural network toolbox. The training algorithm is that of Levenberg–Marquardt, for which the maximum number of epochs is 400 (if early stopping is not triggered). If accuracy on validation set showed no improvement after more than six epochs or the mean squared error (MSE) on the training set is lower than the “goal” we set, the early stopping will be triggered. 

To obtain an optimized BP network, the neuron numbers of first and second hidden layers ($n_{1}$ and $n_{2}$) are selected by the cross-validation method [27]. This method is widely used to select the hyper parameters of ML models. According to this method, the training and validation set is randomly divided into five subsets with same size. For each subset, we use it as the validation set and other four subsets as the training set. Therefore, we can get five prediction results on different validation sets. The mean value of the mean squared error (MSE) of these five results is calculated to optimize the $n_{1}$ and $n_{2}$ [28]. When constructed on the Prairie Grass data set, the BP network with original monitoring parameters obtains the best MSE with $n_{1}=38$ and $n_{2}=4$. With regard to the Indianapolis data set, the best parameter combination is $n_{1}=48$ and $n_{2}=6$.

**Figure 2:**
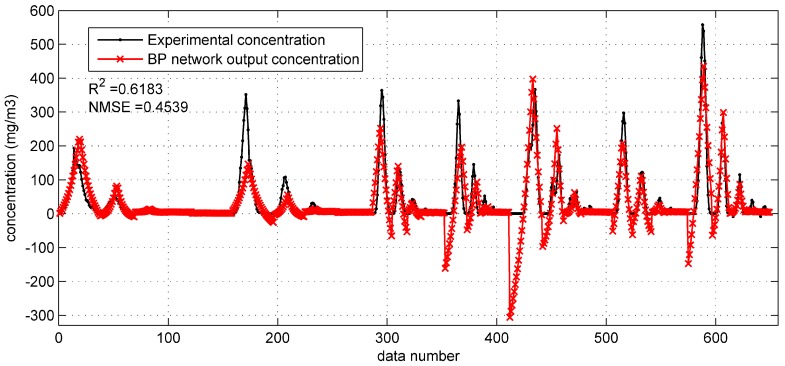
The prediction results of BP network with original monitoring parameters on test set in the Prairie Grass case. NMSE: normalized mean squared error.

When tested on the Prairie Grass test set, the prediction results of the optimized BP network are as shown in Figure 2. The *R*^2^ and the NMSE are applied to evaluate the model performance [29]. They are 0.6183 and 0.4539 respectively, indicating an acceptable accuracy (*R*^2^ is higher than 0.6 and the NMSE is less than 0.5). The relatively high accuracy can also be reflected by the fact that the most predicted concentrations are close to the experimental concentration in this figure. However, there are still some negative concentrations occurring in the prediction, whose values are far from the experimental concentrations. Therefore, the BP network with original parameters should be further improved.

**Figure 3:**
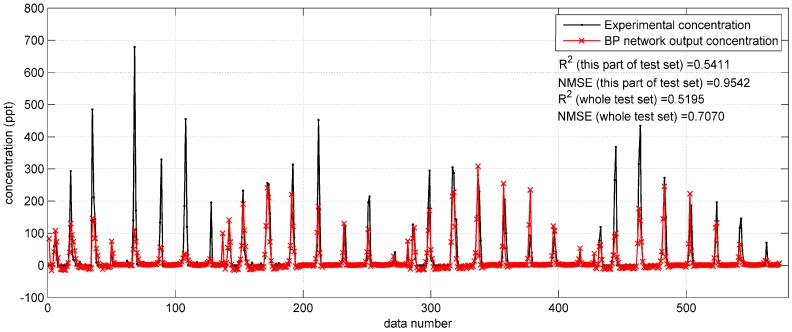
The prediction results of BP network with original monitoring parameters on the test data from 11:00 a.m. to 2:00 p.m. on 17 September (a part of the test set) in the Indianapolis case.

Figure 3 shows the prediction results on a part (only data from 11:00 a.m. to 2:00 p.m., 17 September) of the Indianapolis test set. In this figure, some relatively high concentrations (higher than 200 ppt) are underestimated by the BP network. The $R^{2}$ and NMSE values of the results on the shown part of the test set are 0.5411 and 0.9542, respectively. For the whole test set, the two indicators are 0.5195 and 0.7070, respectively. Obviously, the prediction results are less satisfactory than those in the Prairie Grass case. The less satisfactory performance reflects that the BP network with original parameters is less feasible to predict the tracer dispersion in the Indianapolis case.

#### 3.1.2. BP Network with Integrated Gaussian Input Parameters

To build the BP network with integrated Gaussian parameters, the integrated Gaussian parameters are firstly generated from the original monitoring parameters, and then normalized to form the input vector. The optimized combinations of $n_{1}$ and $n_{2}$ are (58, 8) and (38, 10) for the two field cases, respectively. The details of the training process are the same as the BP network with original parameters, which has been introduced in Section 3.1.1.

**Figure 4:**
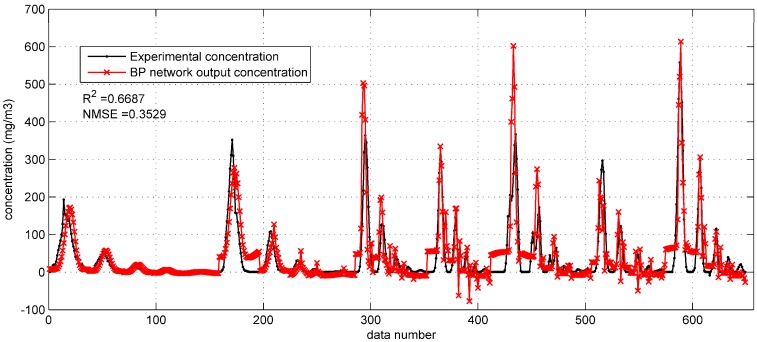
The prediction results of BP network with integrated Gaussian parameters on the test set in the Prairie Grass case.

**Figure 5:**
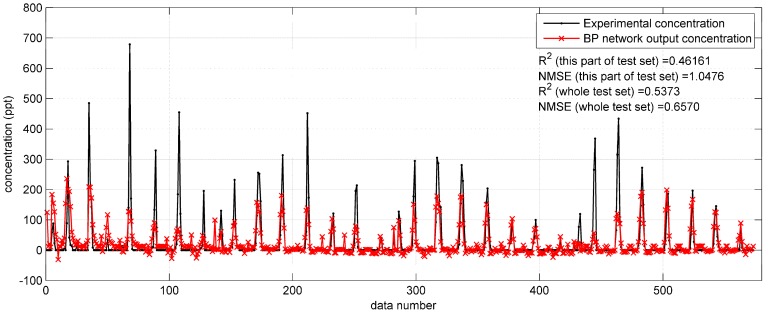
The prediction results of BP network with integrated Gaussian parameters on the data from 11:00 a.m. to 2:00 p.m. on 17 September (a part of the test set) in the Indianapolis case.

Figure 4 and Figure 5 show the prediction results of the optimized BP networks on the test set in the two field cases. In Figure 4, with the higher $R^{2}$ (0.6687) and lower NMSE (0.3529), the BP network with Gaussian parameters yields more accurate predictions than that with original parameters. In addition, the fewer negative concentrations in Figure 4 also indicate the better performance after utilizing the integrated Gaussian parameters. In Figure 5, the $R^{2}$ and NMSE values (the performance indicators) of the whole test set are 0.5373 and 0.6570, respectively, only showing a limited improvement as compared to the $R^{2}$ (0.5195) and NMSE (0.7070) of the BP network in Section 3.1.1. 

The improvement brought about by the Gaussian parameters on the Prairie Grass data set implies that the Gaussian parameters decrease the difficulty of model training. This is because the Gaussian dispersion model is relatively accurate under the flat terrain condition, like the open country of the Prairie Grass field experiment. However, with regard to the reproduction the Indianapolis test set, no significant improvement occurred with Gaussian parameters applied. This difference illustrates that the Gaussian dispersion model is less feasible in the environment of the Indianapolis experiment. The complex topography of the Indianapolis case should be responsible for this difference, which will be analyzed in the Section 3.2.

#### 3.1.3. SVR with Original Monitoring Input Parameters

To build the optimized SVR model, the regression parameter C and spread constant σ are selected by the cross-validation method as well. The SVR model has the smallest MSE when $C=2^{5}$ and $\sigma =2^{-4}$ in the Prairie Grass case, and $C=2^{3}$ and $\sigma =2^{-2}$ in the Indianapolis case, respectively. The construction of the SVR model is conducted by the LIBSVM. The error tolerance ε is set to 0.1. 

**Figure 6:**
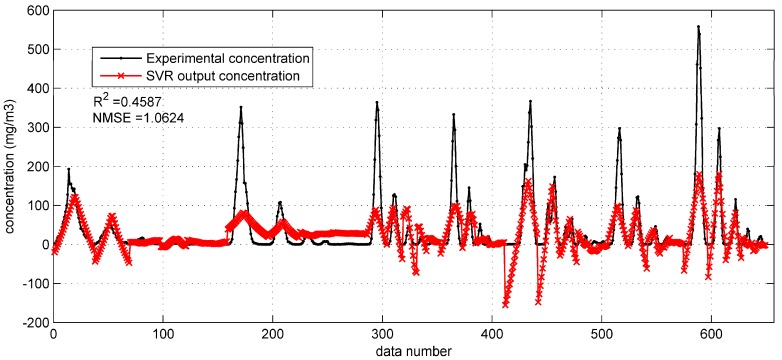
The prediction results of support vector regression (SVR) with original input parameters on the prairie Grass test set.

**Figure 7:**
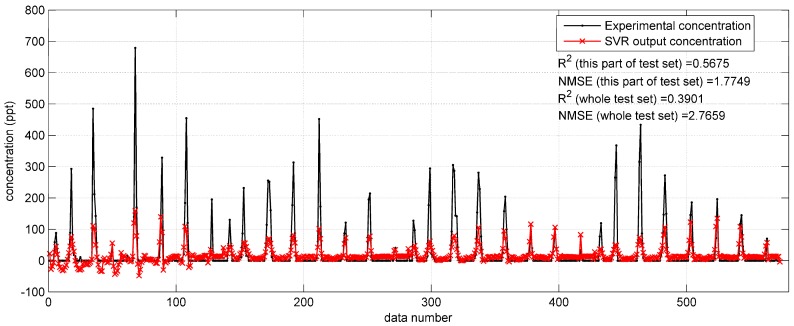
The prediction results of SVR with original input parameters on the data from 11:00 a.m. to 2:00 p.m. on 17 September (a part of the test set) in the Indianapolis case.

Based on the optimized SVR model, the prediction results on the Prairie Grass test set can be obtained as shown in Figure 6. This figure shows that the SVR with original monitoring parameters gets less satisfactory predictions than the BP network with the same inputs (Section 3.1.1). More specifically, the SVR model tends to underestimate the concentration, especially when the experimental data is high. Another problem is that the SVR model also produces some negative concentrations. Therefore, with the relatively low $R^{2}$ (0.4587) and large NMSE (1.0624), this SVR model needs improvement. As for the predictions on Indianapolis test set, they are also terrible ($R^{2}$ = 0.3901, NMSE = 2.7659), as shown in Figure 7. The performances of the SVR model with original monitoring inputs in the two field cases are both far from satisfactory, and poorer than the BP network with the same inputs. This comparison indicates that the SVR model is not as excellent as the BP network in terms of fitting ability.

#### 3.1.4. SVR with Integrated Gaussian Parameters

To improve the SVR with original monitoring parameters, the SVR model with integrated Gaussian parameters is built. The optimized regression parameter C and spread constant σ are determined at ($2^{4}$, $2^{-6}$) and ($2^{2}$, $2^{-1.5}$) for the two field cases, respectively. Other configurations of the training process are the same as the SVR with original parameters (Section 3.1.3). 

**Figure 8:**
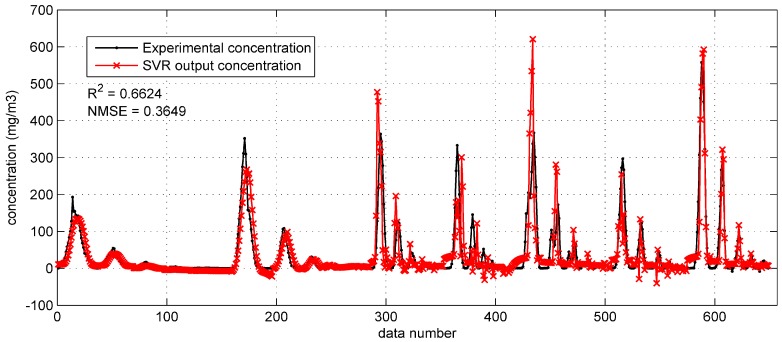
The prediction results of SVR with integrated Gaussian parameters on the Prairie Grass test set.

When applied on the Prairie Grass test set (Figure 8), this SVR model apparently achieves a better performance on reproducing the test data. The two performance indicators are much better ($R^{2}$ = 0.6624, NMSE = 0.3649) compared with the SVR model with original inputs. To be more specific, predictions in Figure 8 approximate the experimental concentration well, even if the observed concentration is high. In addition, fewer negative concentrations appear in the Figure 8. 

**Figure 9:**
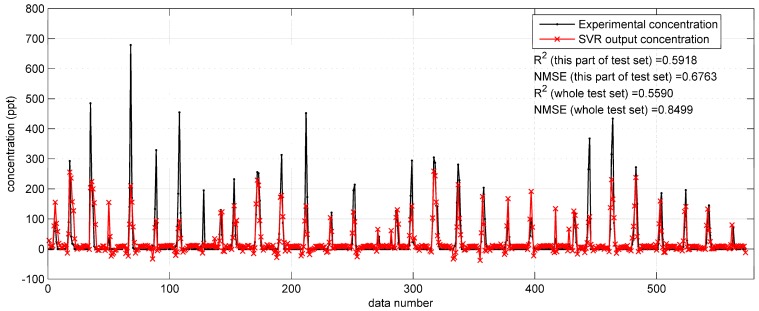
The prediction results of SVR with integrated Gaussian parameters on the data from 11:00 a.m. to 2:00 p.m. on 17 September (a part of the test set) in the Indianapolis case.

With regard to the prediction results on the Indianapolis test set, shown in Figure 9, the $R^{2}$ and NMSE values on the whole test set are 0.5590 and 0.8499, which are better than the indicators ($R^{2}$ = 0.3901, NMSE = 2.7659, Figure 7) of the SVR model with original monitoring inputs. However, the reproduction performance shown in Figure 9 is still not as satisfactory as that in the Prairie Grass case (Figure 8). Therefore, the improvement by the integrated Gaussian parameters is still limited. The reason of this limited improvement continues to be the Gaussian model’s inadaptability to the terrain condition (urban area) of the Indianapolis field experiment.

### 3.2. Results Analysis

The performances of proposed ML prediction models and the Gaussian plume model on test sets of the two field cases are listed in Table 2 and Table 3, respectively. In Table 2, the comparison between the ML models with different input parameters indicates that in the Prairie Grass case, utilizing integrated Gaussian input parameters can improve the prediction performance, especially for the SVR model ($R^{2}$ from 0.4587 to 0.6624, NMSE from 1.0624 to 0.3649). In comparison, Table 3 shows that in the Indianapolis case, the use of integrated Gaussian parameters bring limited improvement to the BP network ($R^{2}$ from 0.5190 to 0.5373, NMSE from 0.7070 to 0.6570). As for the SVR model, the improvement by the Gaussian parameters is more apparent ($R^{2}$ from 0.3901 to 0.5590, NMSE from 2.7659 to 0.8499). However, the SVR model with Gaussian parameters still cannot yield accurate enough predictions as compared with that in Prairie Grass experiment ($R^{2}$: 0.5590 versus 0.6624, NMSE: 0.8499 versus 0.3649). 

The differences between the improvements brought by Gaussian parameters in the two field cases is mainly resulted from the quite different terrain conditions. With almost no obstacle, the terrain of the Prairie Grass field tracer experiment is quite flat. The almost ideal terrain condition means the Gaussian model can describe the hazardous gas dispersion relatively well. This can be indicated by the acceptable performance of the Gaussian plume model in Table 2 ($R^{2}$ = 0.5385, NMSE = 0.7661). Therefore, using integrated Gaussian input parameters helps reduce the difficulty of approximating the input–output function, and improve the prediction performance of ML models. In contrast to the Prairie Grass case, the Indianapolis experiment was implemented on a typical urban area with a number of large buildings. Under this sophisticated terrain condition, the Gaussian model is less capable of modeling the atmospheric dispersion of the tracer. Therefore, the Gaussian parameters only bring limited improvement on the performance of the ML models. In summary, whether the Gaussian parameters can bring improvement depends on its adaptability to the field experiment.

The influence of the terrain conditions can also be reflected by the different performances of ML models in the two field cases. In general, proposed ML models constructed on the Prairie Grass data set (Table 2) outperform those based on the Indianapolis data set (Table 3). In the Indianapolis experiment, a number of large buildings make the tracer concentration distribution more sophisticated than that in the Prairie Grass experiment. Consequently, the difficulty of model training increases, and the performances of ML models in the Indianapolis case deteriorate.

The results also show that the BP network outperforms the SVR model in the fitting ability. It is reflected by the comparison of the BP network and SVR model with the original parameters. With the same inputs, the BP network clearly achieves a better performance than the SVR model in the two field cases. Therefore, this comparison illustrates that the BP network here has a stronger fitting ability to extract the features from the original input parameters and to approximate the input–output function well. In contrast, the poorer $R^{2}$ and NMSE of the SVR model (even worse than the Gaussian plume model in Table 2) indicate that this model encounters the possible under-fitting problem.

As for the computational efficiency, the model building times, prediction times and the total computing times in the two field cases are also listed in the two tables. The model building time represents the duration of the training phase. The total computing time includes the prediction time and the model building time. It is obvious that the Gaussian plume model has the highest efficiency. With regard to other models, their total computing time is acceptable. In addition, it can be seen from the two tables that the greatest computational cost comes from model-building. In contrast, a trained model has fast prediction computation. In terms of conditions, the total computing time of proposed ML models is acceptable, and the computing of prediction is fairly fast. It should be noted that the ML models constructed on the Indianapolis data set has longer building time than the Prairie Grass, because the scale of this data set is larger. In summary, the proposed models can meet the efficiency requirement of the emission management and emergency response.

### 3.3. The Influence of the Training Set and Test Set Scales

Table 2 and Table 3 present the prediction performances of proposed models which are constructed on particular training sets and test sets. In the Prairie Grass case, 60 releases are used for training and 8 releases are for testing, while in the Indianapolis case, 16-day data and 2-day data are applied in the training and test, respectively. As ML models, the performances of proposed models depend on not only the model structure and input selection, but also the training data and test data. Therefore, to further reveal the characteristics (i.e., abilities of fitting and generalization) of proposed ML models, they are constructed on training sets of different scales and evaluated on the remaining test data. Here, the BP network and SVR model with integrated Gaussian parameters are considered. In the Prairie Grass case, the training set size varies from 5 to 67 releases. Therefore, the test set size changes from 63 releases to only one release. In the Indianapolis case, the training set size varies from one day to 16 days, with the test set size changing from 16 days to one day accordingly. For each configuration of training set and test set, the model performances on the two field cases are evaluated. Table A3 and Table A4 in Appendix A display the 32 and 16 different configurations of training set and test set in the two field cases, respectively. Other configurations of the ML models are the same as those optimized models introduced in Section 3.1.

**Figure 10:**
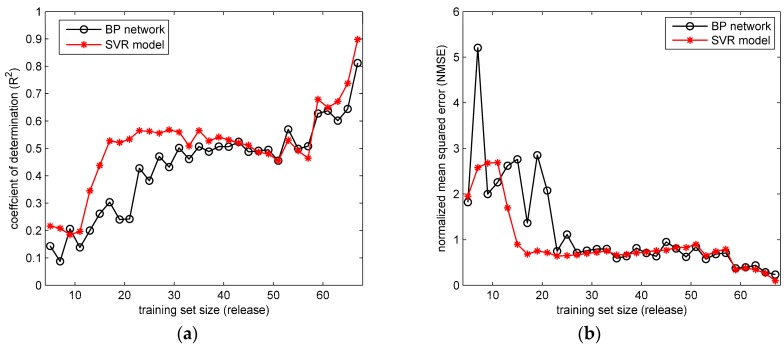
The performances of BP network and SVR model with training sets of different scales in the Prairie Grass case: (**a**) the coefficient of determination ($R^{2}$); (**b**) the normalized mean squared error (NMSE).

**Figure 11:**
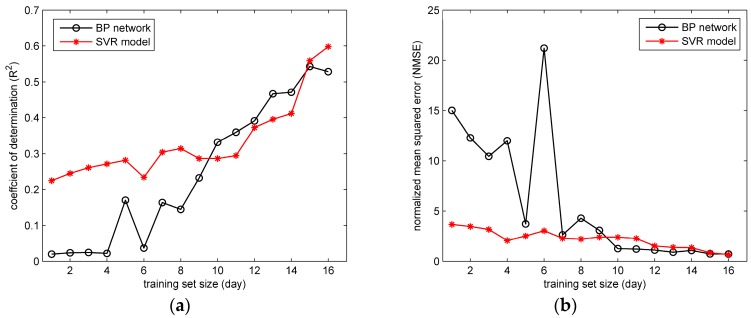
The performances of BP network and SVR model with training sets of different scales in the Indianapolis case: (**a**) the coefficient of determination ($R^{2}$); (**b**) the normalized mean squared error (NMSE).

The prediction results of BP network and SVR models trained by variable-sized training sets are obtained. Figure 10 and Figure 11 show the values of $R^{2}$ and NMSE in the two field cases. It can be seen from the Figure 10 that with the training set scale increasing, the performances of BP network and SVR model both improve (indicated by the rising $R^{2}$ and falling NMSE). These two models both perform well with relatively large training set sizes, especially when the training set has more than 60 releases. Similar trends of $R^{2}$ and NMSE can be found in the Figure 11. The trends of $R^{2}$ and NMSE indicate that the size of training set has a significant impact on the performance of the ML model here. With more training data, the ML model tends to cover more gas dispersion scenarios, and consequently obtain more accurate reproduction results on the test data.

In terms of the comparison between the BP network and SVR model, in Figure 10 the differences between their performances mainly appear when the training set size is relatively small (less than 35 releases). Under this condition, the SVR model apparently achieves higher $R^{2}$ and lower NMSE than BP network. In contrast, the performance of the BP network is unsatisfactory, especially when the training set size is smaller than 23 releases (most values of $R^{2}$ are lower than 0.4, and NMSE are larger than 1.0). Similarly, in Figure 11 the SVR model with Gaussian parameters shows a better performance on $R^{2}$ and NMSE than the BP network when the training set size is smaller than 10 days. This comparison indicates that the performance of SVR model with Gaussian parameters is less dependent on the training data scale, which implies a better ability of generalization. The better generalization results from the optimization goal of the SVR model. Instead of minimizing the training error, the SVR attempts to minimize the generalization error bound (the loss function parameter ε) so as to achieve a generalized performance [30]. Therefore, the SVR model yields more accurate predictions on the test set under the condition of small-sized training sets. As for the BP network, the unsatisfactory results under the small-scale training sets reflect the possible over-fitting problem. When over-fitting the training data, the BP network tends to reproduce the training set “too accurately”, which means the model is only valid on the training set. Therefore, when applied in the test data (which is usually quite different from the training data), the BP network sometimes obtains unsatisfactory results, especially when the training set scale is limited. Therefore, considering all the experiment results, the SVR has a better ability for generalization, but tends to encounter the under-fitting problem when the input–output relationship is quite complex. In contrast, the BP network has an excellent fitting ability, but it is likely to over-fit the training data, and achieve unsatisfactory generalization especially when the scale of training set is relatively small. These characteristics are valuable for the prediction model selection in field cases.

## 4. Discussion

The results analysis shows that the Gaussian parameters method is case-sensitive, and whether the Gaussian parameters can bring improvement depends on its adaptability to the field experiment. Therefore, specifying the application condition or the application case of the Gaussian parameters method is useful to the user of our ML prediction models. The performance of the Gaussian parameters method depends on the adaptability of the Gaussian plume model to the field case. An ideal environment for the Gaussian plume model should meet some conditions. Firstly, the terrain should be open and flat, and the surface property should be uniform. Then, the Gaussian plume model is accurate in the near-field case where the dispersion range is usually less than 10 km. Moreover, the Gaussian plume model does not take the chemical reaction of the dispersion material into consideration. Finally, the Gaussian plume model requires the wind field to be stable and uniform, because this model uses the mean value of the wind field to calculate concentration. When a field case satisfies these conditions well, the Gaussian plume model and the ML model with Gaussian parameters tend to have high accuracy accordingly. 

The ML model provides an effective method to predict the hazardous gas dispersion in a particular field case. Compared with the deterministic atmospheric dispersion model (e.g., the Gaussian model), the ML model can adjust itself according to the training data, and tend to produce more reliable predictions. Therefore, the ML model is suitable for the hazardous gas dispersion prediction in the field case, especially for complex field experiments (e.g., the urban area). However, this characteristic is also the limit of the ML model. That is the ML prediction model is valid only in the situations where it is developed. The ability to export the results of a particular situation to others is dependent on the similarity of the these situations. For example, the ML model developed from the Prairie Grass data set is clearly not feasible on the Indianapolis data set, because the two field tracer experiments were conducted in quite different terrain conditions. Therefore, a new ML model for the Indianapolis case should be built.

The results of the two field cases reflect the differences between BP network and SVR model on the abilities of fitting and generalization. The SVR model has a better ability of generalization but tends to under-fit the training data sometimes. In contrast, the BP network shows a better fitting ability but encounters the over-fitting problem more easily. Therefore, how to overcome the drawbacks of the two models is valuable for improving the model performance. The hybrid model combining the advantages of both models may be a feasible alternative. Further, the characteristics of the two ML models provide some guidance for the model selection in the hazardous gas dispersion prediction. When the scale of observed data is limited or the input–output relationship is not so complex, the SVR is a better choice due to its good generalization. If the observation size is large enough to cover most scenarios, or the function to be fitted is quite complex, the BP network is better because of its excellent fitting ability.

Moreover, there are still several measures to further improve the performances of proposed ML models, especially in the Indianapolis case. First, more observed data is likely to contribute to the model accuracy. Although the Indianapolis data set has more than 20,000 samples of the tracer, more than three quarters of these samples are zero measurements, which are almost meaningless. Therefore, more valid observations are necessary. In addition, some more sophisticated parameters can be considered in the model construction. For example, the LS or CFD model may be able to accurately describe the tracer dispersion in air in the Indianapolis case. Therefore, the LS-related or CFD-related parameters may possibly help to improve model accuracy.

In Section 3.2 (Results Analysis), we attribute the different model performances in the two field cases to the different topography. In fact, besides the terrain condition, there are still other factors that influence the atmospheric dispersion potentially. These factors include the tracer type (SO_2_ and SF_6_), the measurement distance and time, etc. For example, SO_2_ and SF_6_ have different chemical properties in air. Therefore, their lifetimes in the atmospheric dispersion may be different, causing the difference in the concentration observations. In addition, the measurement distance in the Prairie Grass case is closer to the source. This may lead to the more satisfactory performance of the ML models with Gaussian parameters, because the Gaussian model is more accurate in the near field case. Therefore, in the future work, we will analyze the impact of other factors on the atmospheric dispersion, and give a more clear explanation of the model performances in the two field cases.

## 5. Conclusions

This paper compares two machine learning models (i.e., the BP network and SVR) for hazardous gas dispersion prediction in field cases. These ML models are built firstly by original input parameters. Then, in order to enhance the prediction accuracy, the integrated Gaussian parameters are formed by the original monitoring parameters, and used as the inputs of the ML models. The two ML models with different inputs are tested and compared using two typical field data sets. Further, the influence of the training set scale is analyzed as well. 

Results illustrate that the performances of proposed ML models in the Prairie Grass data set are more satisfactory than those of the Indianapolis case generally, because the Indianapolis field experiment involves complex terrain conditions. As for the input selection, the use of the integrated Gaussian parameters indeed improves prediction accuracy. However, the extent of the improvement relies on the adaptability of the Gaussian model to the field experiment. In terms of the comparison of the two ML models, the BP network usually has a stronger fitting ability, while the SVR model achieves a better generalization. This comparison can help researchers and managers to select models for hazardous gas dispersion prediction.

In conclusion, the proposed ML models provide an effective way of predicting hazardous gas dispersion in field cases. With relatively high prediction accuracy, these ML models will provide strong support for the management and emergency response to hazardous gas emissions and accidental leaks. However, under complex terrain conditions (like the Indianapolis case) the prediction accuracy of the proposed ML models still needs improvement. Future work will focus on the further improvement of the ML models, especially in some complex field cases. For example, the LS or CFD-related parameters can be used to enhance the prediction accuracy of the ML models. In addition, besides the terrain condition, other factors such as the tracer type, and the measurement distance and time will be considered to explain the atmospheric dispersion in the two field cases more clearly.

## Figures and Tables

**Table 1:** Common original monitoring parameters in the Prairie Grass data set.

Parameters	Symbol	Unit
Downwind distance	$D_{x}$	m
Crosswind distance	$D_{y}$	m
Source strength	Q	g·s^−1^
Wind speed	V	m·s^−1^
Wind direction	Dir	deg
Atmospheric stability class	STA	/
Air temperature	T	°C
Source height	$H_{s}$	m
Target height	Z	m
Mixing height	$Z_{m}$	m
Cloud height	$Z_{c}$	m
Standard deviation of wind direction	$\sigma_{d}$	deg
Relative humidity	RH	%

**Table 2:** Prediction performances of proposed machine learning (ML) models on the Prairie Grass test set.

Methods	*R* ^2^	NMSE	Model Building Time (s)	Prediction Time (s)	Total Computing Time (s)
Gaussian plume model	0.5385	0.7661	/	1.698 × 10^−3^	1.698 × 10^−3^
BP network with original parameters	0.6183	0.4539	6.773	7.734 × 10^−3^	6.781
BP network with Gaussian parameters	0.6687	0.3529	4.413	8.21 × 10^−3^	4.421
SVR with original parameters	0.4587	1.0624	2.429	6.277 × 10^−2^	2.492
SVR with Gaussian parameters	0.6624	0.3649	3.967	5.009 × 10^−2^	4.017

**Table 3:** Prediction performances of proposed ML models on the Indianapolis test set.

Methods	*R* ^2^	NMSE	Model Building Time (s)	Prediction Time (s)	Total Computing Time (s)
Gaussian plume model	0.1018	6.4274	/	1.219 × 10^−2^	1.219 × 10^−2^
BP network with original parameters	0.5190	0.7070	27.431	4.086 × 10^−2^	27.472
BP network with Gaussian parameters	0.5373	0.6570	31.705	3.940 × 10^−2^	31.744
SVR with original parameters	0.3901	2.7659	75.098	1.030	76.128
SVR with Gaussian parameters	0.5590	0.8499	95.532	1.372	96.904

## Appendix A

Table A1 and Table A2 show the statistical indicators of the input parameters before normalization in the two field cases.

**Table A1:** The statistical indicators of the inputs in the Prairie Grass case.

Input Parameter	Symbol	Maximum	Minimum	Mean	Standard Deviation
Downwind distance 1 (m)	$D_{x1}$	800	−470.228	283.629	284.360
Downwind distance 2 (m)	$D_{x2}$	800	−545.599	279.037	283.309
Downwind distance 3 (m)	$D_{x3}$	800	−363.192	285.652	283.113
Downwind distance 4 (m)	$D_{x4}$	800	−575.472	278.641	284.305
Crosswind distance 1 (m)	$D_{y1}$	800	0	61.923	101.207
Crosswind distance 2 (m)	$D_{y2}$	800	0	72.617	109.474
Crosswind distance 3 (m)	$D_{y3}$	800	0	62.555	99.218
Crosswind distance 4 (m)	$D_{y4}$	800	0	70.914	109.015
Source strength (g·s^−1^)	Q	104.1	38.5	77.983	25.591
Wind speed 1 (m·s^−1^)	$V_{1}$	9	1.25	4.909	2.069
Wind speed 2 (m·s^−1^)	$V_{2}$	9.26	1.65	4.987	2.079
Wind speed 3 (m·s^−1^)	$V_{3}$	8.52	1.37	4.862	2.003
Wind speed 4 (m·s^−1^)	$V_{4}$	9.36	1.65	4.945	2.065
Wind direction 1 (deg)	$Dir_{1}$	245	128	186.473	26.363
Wind direction 2 (deg)	$Dir_{2}$	237	105	191.518	25.259
Wind direction 3 (deg)	$Dir_{3}$	244	128	185.580	25.411
Wind direction 4 (deg)	$Dir_{4}$	243	109	191.800	26.241
Air temperature (°C)	T	35.58	15.347	26.0411	5.753
Gaussian parameter (y) 1	$G_{y1}$	5.233 × 10^11^	0	2.521 × 10^10^	6.206 × 10^10^
Gaussian parameter (y) 2	$G_{y2}$	5.233 × 10^11^	0	2.361 × 10^10^	6.182 × 10^10^
Gaussian parameter (y) 3	$G_{y3}$	5.233 × 10^11^	0	2.500 × 10^10^	5.825 × 10^10^
Gaussian parameter (y) 4	$G_{y4}$	5.233 × 10^11^	0	2.375 × 10^10^	5.952 × 10^10^
Gaussian parameter (z) 1	$G_{z1}$	8.093 × 10^11^	0	1.736 × 10^11^	1.493 × 10^11^
Gaussian parameter (z) 1	$G_{z2}$	8.102 × 10^11^	0	1.751 × 10^11^	1.482 × 10^11^
Gaussian parameter (z) 1	$G_{z3}$	8.102 × 10^11^	0	1.617 × 10^11^	1.391 × 10^11^
Gaussian parameter (z) 1	$G_{z4}$	8.102 × 10^11^	0	1.679 × 10^11^	1.444 × 10^11^

**Table A2:** The statistical indicators of the inputs in the Indianapolis case.

Input Parameter	Symbol	Maximum	Minimum	Mean	Standard Deviation
Downwind distance 1 (m)	$D_{x1}$	1.268 × 10^4^	−1.105 × 10^4^	1.517 × 10^3^	2.705 × 10^3^
Downwind distance 2 (m)	$D_{x2}$	1.266 × 10^4^	−1.200 × 10^4^	1.211 × 10^3^	2.876 × 10^3^
Downwind distance 3 (m)	$D_{x3}$	1.265 × 10^4^	−1.210 × 10^4^	1.364 × 10^3^	2.804 × 10^3^
Downwind distance 4 (m)	$D_{x4}$	1.270 × 10^4^	−1.255 × 10^4^	1.400 × 10^3^	2.756 × 10^3^
Crosswind distance 1 (m)	$D_{y1}$	1.273 × 10^4^	0.075	2.166 × 10^3^	2.303 × 10^3^
Crosswind distance 2 (m)	$D_{y2}$	1.267 × 10^4^	0.069	2.145 × 10^3^	2.296 × 10^3^
Crosswind distance 3 (m)	$D_{y3}$	1.271 × 10^4^	0.172	2.150 × 10^3^	2.295 × 10^3^
Crosswind distance 4 (m)	$D_{y4}$	1.272 × 10^4^	0.106	2.173 × 10^3^	2.309 × 10^3^
Source strength (g·s^−1^)	Q	4.670	4.600	4.656	0.011
Wind speed 1 (m·s^−1^)	$V_{1}$	11.150	0.640	4.932	2.106
Wind speed 2 (m·s^−1^)	$V_{2}$	5.910	0.520	2.741	1.110
Wind speed 3 (m·s^−1^)	$V_{3}$	7.130	0.270	2.637	1.619
Wind speed 4 (m·s^−1^)	$V_{4}$	5.800	0.400	2.401	1.225
Wind direction 1 (deg)	$Dir_{1}$	319	5	189.837	61.896
Wind direction 2 (deg)	$Dir_{2}$	336	13	188.355	66.071
Wind direction 3 (deg)	$Dir_{3}$	354	4	190.091	61.599
Wind direction 4 (deg)	$Dir_{4}$	327	22	186.145	63.399
Gaussian parameter (y) 1	$G_{y1}$	1.369 × 10^11^	0	4.450 × 10^8^	2.888 × 10^9^
Gaussian parameter (y) 2	$G_{y2}$	1.051 × 10^11^	0	4.226 × 10^8^	2.633 × 10^9^
Gaussian parameter (y) 3	$G_{y3}$	1.369 × 10^11^	0	4.618 × 10^8^	2.982 × 10^9^
Gaussian parameter (y) 4	$G_{y4}$	1.369 × 10^11^	0	4.685 × 10^8^	3.150 × 10^9^
Gaussian parameter (z) 1	$G_{z1}$	1.448 × 10^11^	0	6.463 × 10^9^	5.319 × 10^9^
Gaussian parameter (z) 1	$G_{z2}$	1.448 × 10^11^	0	5.982 × 10^9^	5.382 × 10^9^
Gaussian parameter (z) 1	$G_{z3}$	1.448 × 10^11^	0	6.217 × 10^9^	5.350 × 10^9^
Gaussian parameter (z) 1	$G_{z4}$	1.448 × 10^11^	0	6.259 × 10^9^	5.350 × 10^9^

The Table A3 and Table A4 describe the details of 32 and 16 different configurations of the training set and test set in the two field cases.

**Table A3:** The configurations of the training sets and test sets in the Prairie Grass case.

Configuration ID	Training Set Size (Release)	Percentage of the Training Set	Test Set Size (Release)	Percentage of Test Set
1	5	8.43%	62	91.57%
2	7	12.21%	60	87.79%
3	9	16.46%	59	83.54%
4	11	20.12%	57	79.88%
5	13	23.08%	55	76.92%
6	15	27.26%	53	72.74%
7	17	30.44%	51	69.56%
8	19	33.26%	49	66.74%
9	21	36.09%	47	63.91%
10	23	38.24%	45	61.76%
11	25	42.62%	43	57.38%
12	27	46.89%	41	53.11%
13	29	47.97%	39	52.03%
14	31	51.90%	37	48.10%
15	33	54.52%	35	45.48%
16	35	56.91%	33	43.09%
17	37	59.10%	31	40.90%
18	39	61.16%	29	38.84%
19	41	63.36%	27	36.64%
20	43	67.23%	25	32.77%
21	45	71.30%	23	28.70%
22	47	74.88%	21	25.12%
23	49	78.72%	19	21.28%
24	51	82.90%	17	17.10%
25	53	83.58%	15	16.42%
26	55	85.54%	13	14.46%
27	57	88.33%	11	11.67%
28	59	89.62%	9	10.38%
29	61	92.87%	7	7.13%
30	63	95.69%	5	4.31%
31	65	96.54%	3	3.46%
32	67	98.91%	1	1.09%

**Table A4:** The configurations of the training sets and test sets in the Indianapolis case.

Configuration ID	Training Set Size (Day)	Percentage of the Training Set	Test Set Size (Day)	Percentage of Test Set
1	1	5.95%	16	94.05%
2	2	12.15%	15	87.85%
3	3	18.74%	14	81.26%
4	4	24.45%	13	75.55%
5	5	30.41%	12	69.59%
6	6	36.87%	11	63.13%
7	7	41.97%	10	58.03%
8	8	47.76%	9	52.24%
9	9	54.10%	8	45.90%
10	10	60.21%	7	39.79%
11	11	65.92%	6	34.08%
12	12	71.86%	5	28.14%
13	13	77.46%	4	22.54%
14	14	83.46%	3	16.54%
15	15	89.02%	2	10.98%
16	16	94.58%	1	5.42%
